# Advanced research trends in dye-sensitized solar cells

**DOI:** 10.1039/d1ta00690h

**Published:** 2021-03-10

**Authors:** Mikko Kokkonen, Parisa Talebi, Jin Zhou, Somayyeh Asgari, Sohail Ahmed Soomro, Farid Elsehrawy, Janne Halme, Shahzada Ahmad, Anders Hagfeldt, Syed Ghufran Hashmi

**Affiliations:** Microelectronics Research Unit, Faculty of Information Technology & Electrical Engineering, University of Oulu P. O. Box 4500 FI-90014 Finland ghufran.hashmi@oulu.fi; Nano and Molecular Systems Research Unit, University of Oulu FIN-90014 Finland; Optoelectronics and Measurement Techniques Research Unit, Faculty of Information Technology and Electrical Engineering, University of Oulu Oulu Finland; Center for Ubiquitous Computing, Department of Information Technology and Electrical Engineering, University of Oulu Finland; New Energy Technologies Research Group, Department of Applied Physics, Aalto University P.O. Box 15100 FI-00076 Aalto Finland; BCMaterials-Basque Center for Materials, Applications and Nanostructures UPV/EHU Science Park 48940 Leioa Spain; IKERBASQUE, Basque Foundation for Science Bilbao 48009 Spain; Department of Chemistry, Ångström Laboratory, Uppsala University P. O. Box 523 75120 Uppsala Sweden

## Abstract

Dye-sensitized solar cells (DSSCs) are an efficient photovoltaic technology for powering electronic applications such as wireless sensors with indoor light. Their low cost and abundant materials, as well as their capability to be manufactured as thin and light-weight flexible solar modules highlight their potential for economic indoor photovoltaics. However, their fabrication methods must be scaled to industrial manufacturing with high photovoltaic efficiency and performance stability under typical indoor conditions. This paper reviews the recent progress in DSSC research towards this goal through the development of new device structures, alternative redox shuttles, solid-state hole conductors, TiO_2_ photoelectrodes, catalyst materials, and sealing techniques. We discuss how each functional component of a DSSC has been improved with these new materials and fabrication techniques. In addition, we propose a scalable cell fabrication process that integrates these developments to a new monolithic cell design based on several features including inkjet and screen printing of the dye, a solid state hole conductor, PEDOT contact, compact TiO_2_, mesoporous TiO_2_, carbon nanotubes counter electrode, epoxy encapsulation layers and silver conductors. Finally, we discuss the need to design new stability testing protocols to assess the probable deployment of DSSCs in portable electronics and internet-of-things devices.

## Abstract 2

Dye-sensitized solar cells (DSSCs) are an efficient photovoltaic technology for powering electronic applications such as wireless sensors with indoor light.

## Graphical abstract

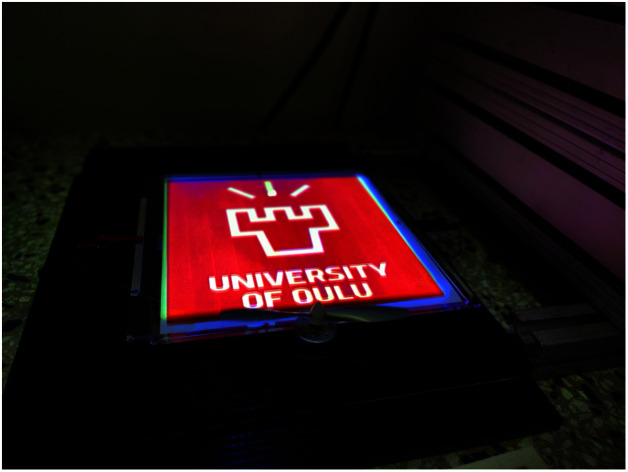

## 1. Introduction

As the global population continues to increase, the resulting energy demands have escalated, along with concerns about greenhouse gas emissions and climate change. These have greatly motivated researchers worldwide to search for alternative and clean methods of energy production. Among the various renewable energy sources, solar energy offers abundant, silent and eco-friendly power that has enormous potential for meeting the global energy consumption demands.^1,2^ Photovoltaics (PV)^3^ provides an opportunity to affordably convert this abundant and clean energy source into electrical energy.

Of the PV technologies, crystalline silicon (Si)-based PV systems have dominated the global PV market over the past five decades. This is largely because of their beneficial features such as efficient electricity generation under full sunlight, good photovoltaic performance stability in all climatic conditions, as well as the maturity around their research and development (R&D) activities and associated material value chain. Nevertheless, there are several drawbacks associated with Si-based PV systems, including their energy intensive production processes, poor aesthetics, and low photovoltaic performance in low light intensities. Together these have limited their widespread use in building integrated photovoltaics (BIPV), portable electronics and indoor applications.^2–5^

In contrast to traditional PV techonologies, third-generation photovoltaic technologies such as dye-sensitized solar cells (DSSCs),^6^ organic solar cells (OSCs)^7^ and perovskite solar cells (PSCs)^8,9^ have been developed using low-cost and abundant materials with facile and scalable fabrication methods. Currently, their lower solar-to-electrical energy conversion efficiency and photovoltaic performance stability has prevented them from successfully competing with the existing commercial PV technologies for bulk electricity generation outdoors.^10,11^ However, their capability to be manufactured as thin and light-weight flexible solar modules^12,13^ make them ideal for portable electronics.^14^ Similarly, their high efficiency under dim light – which outperforms other existing technologies at typical indoor conditions – makes them promising for ambient energy harvesting for the wireless sensors used in the internet of things (IoT) devices.^5,14–16^

**Fig. 1:**
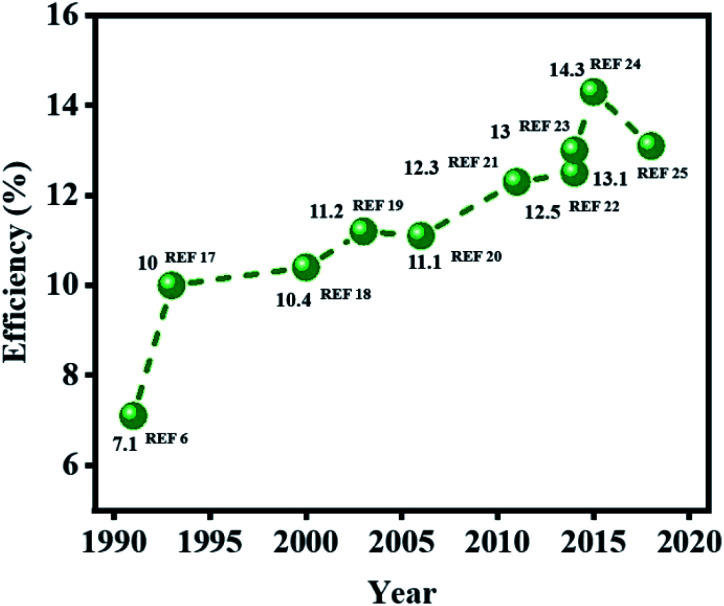
Evolution of conversion efficiencies of DSSCs in recent years.

This review highlights the recent progress in developing new materials for producing high performance DSSC-based photovoltaic devices (Fig. 1).^6,17–25^ New DSSC device designs that have appeared in recent years using alternative redox shuttles and catalyst materials are described, along with the new opportunities for their possible integration in portable electronics, wireless sensor network and IoT devices.^5^ This review also compiles the progress made in the associated materials, revealing how each functional component of a DSSC has been improved with alternative materials and fabrication procedures. Moreover, a strategy to produce a novel cell design is also suggested, which may be achieved in the near future by using scalable fabrication methods. New sealing methods to produce stable DSSC devices, as well as their advantages and limitations, are also discussed. Finally, the possibility to design new stability testing protocols to assess the probable deployment of DSSCs in portable electronics and IoT devices are addressed, along with their future direction. Overall, this review provides the most up-to-date viewpoints on the latest research trends that have emerged during the development of the next generation of DSSC technology.

## 2. Novel device designs

**Fig. 2:**
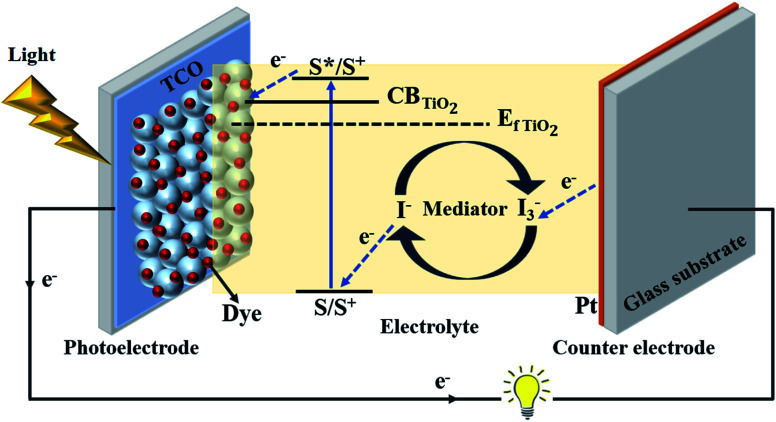
Schematic illustration representing device structure and working principle of a dye-sensitized solar cell. CB = conduction band, *E*_f TiO2_ = fermi level of TiO_2_, S = ground state of dye sensitizer molecule, S* = excited state of dye sensitizer molecule, S^0^ = oxidized dye, S^+^ = charge separation, I^−^ = iodide ion and I_3_^−^ = triiodide ion.

The traditional embodiment of a dye-sensitized solar cell (DSSC) utilizes two transparent conducting oxide (TCO) coated glass electrodes, usually fluorine doped tin oxide (FTO) coated glass substrates (Fig. 2).^26^ One of these glass substrates is covered with an interconnected TiO_2_ particle-based nanocrystalline layer, 10–15 μm thick, which serves as a photoelectrode (PE) when sensitized with a dye – typically a ruthenium (Ru)-based organometallic molecule. A second glass substrate, coated with a catalyst (such as Pt), serves as a counter electrode (CE). The PE and CE are either laminated together with a 10–45 μm thick thermoplastic spacer foil, or are separated by a thick (1–30 μm) and porous insulator (*e.g.* ZrO_2_- or Al_2_O_3_-based insulating layers)^27^ to avoid a short circuit between them.^26^

**Fig. 3:**
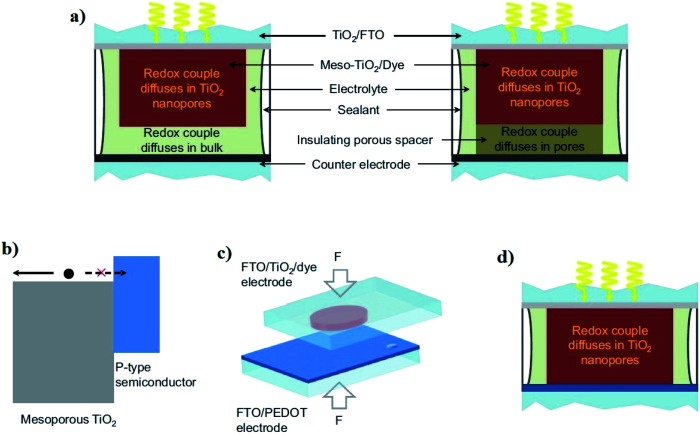
(a) Traditional DSSC use either a thermoplastic or porous insulating spacer to avoid short circuit between the mesoporous TiO_2_ and the counter electrode. (b) Type II junction alignment^31^ of the band edges for the mesoporous TiO_2_ film and a p-type semiconductor layer. The p-type semiconductor serves as an electron-blocking hole-selective charge collection layer. (c) The sensitized TiO_2_ electrode and the PEDOT semiconductor-based counter electrode make direct contact *via* mechanically pressing and make a new DSSC embodiment. (d) In the DSSC with the contacted electrodes, the redox couple diffuses merely through the mesoscopic TiO_2_ film (reproduced from reference with permission^25^).

During the operation of the cell, charges get exchanged between PE and CE through liquid electrolyte containing redox mediator (typically iodide/triiodide-based redox shuttles). The mediator does not only diffuse in the porous TiO_2_ electrode but also through the porous spacer and through the bulk phase of the liquid electrolyte (Fig. 3a).^25^ Hence, the thickness of the thermoplastic and spacer directly influences the photovoltaic performance of DSSCs through mass transport and the diffusion resistance (*R*_D_) of the bulk electrolyte.^25,26,28^ Presently, minimizing *R*_D_ has only been realized either by adjusting the thermoplastic, adjusting the porous insulator thicknesses,^28,29^ or by using low viscosity solvent-based electrolytes to produce high efficiency DSSCs.^23,30^

In this regard, Cao *et al.* recently reported a unique DSSC in which the mesoporous TiO_2_-based PE and poly(3,4-ethylenedioxythiophene; PEDOT) catalyst-based CE were in physical contact without using any spacer in between them (Fig. 3b–d). Interestingly, the fabricated DSSCs did not exhibit electronic shunting after the electrolyte was injected into the cell channel (to achieve charge transport between the contacted electrodes). Instead, it formed a type II junction^31^ in which the sensitized semiconducting oxide (TiO_2_) layer was used as an n-type inorganic semiconductor, and the PEDOT layer served as a hole-selective electron-blocking layer-based p-type polymer semiconductor.^25^ As a result, the *R*_D_ of the contacted DSSCs was suppressed, which consequently contributed to the enhancement of their photovoltaic performance. An impressive solar-to-electrical conversion efficiency (13.1%) was demonstrated with alternative copper (Cu) redox shuttle-based liquid electrolytes and co-sensitized TiO_2_ electrodes (with dyes Y123 and XY1b) under full sunlight illumination. More interestingly, the same devices exhibited very high (32%) conversion efficiency and 101 mW cm^−2^ maximal output power density under artificial indoor lighting (1000 lux), which is promising for energizing sensors, IoT devices and portable electronics.^25^

Recently, the conversion efficiencies of similar spacer-free DSSCs have further improved to 34.0%, 32.7% and 31.4% under 1000, 500 and 200 lux of fluorescent light, respectively.^5^ By forming an array of serially connected DSSCs, these were demonstrated to energize both IoT nodes and a base station under 1000 lux produced by a fluorescent lamp, thus highlighting the potential for their future contributions in a data driven economy – which is envisioned to be governed through smart autonomous systems and IoT devices.^5^

Similar to spacer-free DSSCs, the so-called solid-state Zombie Cells (named after the discovery that so-called “dead” DSSCs, that had lost their electrolyte solvent due to leakage, unexpectedly were still generating electricity)^32^ are also demonstrated in the work of Michaels *et al.*^5^ Specifically, the electrolyte was intentionally dried out by evaporating its solvent through drilled holes on a glass electrode, leaving the Cu-based redox pair and additives to act as a solid hole transport material in the cell channel. This contributed to impressive conversion efficiencies (10.2%, 11.2% and 30%) when tested under AM 1.5G simulated sunlight, 10% sunlight and 1000 lux illumination conditions, respectively.^5^

Despite this promising evidence, the present limitations associated with these advanced DSSCs include: (1) very thick (6–8 mm) device architecture due to two glass substrates, which may limit their efficient integration in light harvesting and power generation units for IoT devices. (2) High cost of using two glass substrates, which keeps momentous share among all the materials in DSSC manufacturing.^29,33,34^ (3) The approach for creating a Cu redox and additive-based solid hole transport layer in between the PE and CE is unrealistic, as the solvent of the Cu electrolyte needs to be evaporated for a prolonged period (72–96 h); this is impractical for rapid batch production.^5^ (4) The drilled holes, which cause an increase in the non-active area, overall cell resistance (*R*_CELL_) and overall production cost since an additional thermoplastic and glass cover or UV glue is needed to close them.^5,25^ (5) The chemical robustness of the PEDOT catalyst is rarely reported and hence needs further investigation, since it was recently suspected to cause electrolyte degradation under a 100 mW cm^−2^ illumination-based light soaking test.^35^

**Fig. 4:**
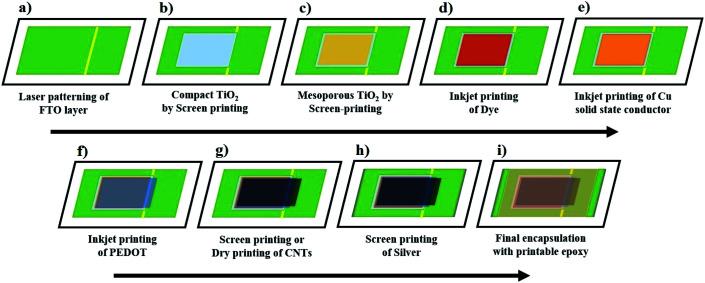
Proposed process flow for producing advanced monolithic DSSCs with alternative Cu redox shuttles-based electrolytes and solid hole conductors.

In light of all these challenges, Fig. 4 outlines a process flow that could allow fabrication of the Cu redox and PEDOT electrode based DSSCs in a monolithic cell configuration through scalable fabrication methods such as screen-printing or inkjet printing. Such monolithic DSSC device design may not only influence the overall production cost through integrating active layers on a single glass substrate, but it may also provide a possibility for further reducing the cell resistance, for example by eliminating the drilled holes and spacer layer or channel produced by an insulator layer or a thermoplastic sealant, respectively. These may not be achieved in the conventional double glass-based DSSC device geometry.^5,32^

The proposed process of making this monolithic cell could begin by first laser patterning the TCO glass substrates into the anode and cathode of the aimed solar cell (Fig. 4a).

Next, the hole-blocking compact TiO_2_ layer can be screen-printed on the anode substrate using commercially available screen-printable pastes (Fig. 4b).^36,37^ The process continues with screen-printing a mesoscopic semiconducting oxide (TiO_2_) layer for electron transport (Fig. 4c), followed by its staining with a concentrated dye solution through inkjet printing (Fig. 4d).^38^

Contrary to the traditional liquid electrolyte filling method *via* drilled holes,^5^ the Cu redox-based liquid or solid-state hole conductors may be directly printed over sensitized TiO_2_ layers (Fig. 4e).^39^ This may decrease the non-active area and can also minimize the overall cell resistance.^39^

Using commercially available inks,^40,41^ the p-type semiconducting polymer and catalyst (*i.e.* PEDOT) layer may also be unconventionally printed directly over the printed Cu redox hole conductor (Fig. 4f) instead of following the traditional electro-polymerization method.

Similarly, the conductive carbonaceous electrodes may further be produced over the PEDOT layer (Fig. 4g) either by screen-printing^42^ or dry printing-based techniques.^43,44^ This would promote both the conductivity and efficient hole collection.

Next, the metal contacts can be produced by screen-printing a silver paste at the edges (Fig. 4h), whereas a screen-printable epoxy^45^ may be chosen as the final step for protecting the active layers (Fig. 4i).

The suggested process sequence may potentially transform the present two glass substrate DSSC into an advanced, fully printable and monolithic device that may exhibit higher photovoltaic performance with accurate and reliable process control – which has been considered as a key factor for the large-scale manufacturing of any solar cell technology.^46,47^ Although fabricated on a rigid glass substrate, these monolithic DSSCs would be cheaper to produce and thinner than the present two glass substrate based DSSCs, and therefore potentially easier to integrate into energy harvesting applications.

Also, contrary to traditional charge-transport materials (CTMs)^48^ such as Spiro-OMeTAD, which have been widely used for both solid-state DSSCs and emerging Perovskite Solar Cells (PSCs) based technologies,^49,50^ the abundant Cu-redox based solid hole transport material may also contribute to additional cost reductions, which can be accounted as a complementary feature in addition to their active functioning to produce DSSC devices with high efficiency.

## 3. Improved photovoltaic performance with alternative redox shuttles

Along with the device designs, electrolyte formulations with alternative redox shuttles have also shown remarkable progress recently and have significantly increased the energy conversion efficiency of DSSCs.^21,23,25,30,51,52^

Although they are known for their impressive conversion efficiencies and robust long-term stability,^20,53,54^ traditional iodide/triiodide redox shuttle-based electrolytes have been swiftly replaced with alternative redox couples to overcome their intrinsic bottlenecks *i.e.* their lower redox potential or their corrosive nature to metal fingers, which limit the photovoltaic performance stability of the fabricated DSSC.^11^

Among the numerous alternatives^52,55,56^ cobalt (Co) and copper (Cu) redox shuttle-based electrolytes have received considerable attention. This is largely because many of their unique characteristics have been revealed, such as rapid dye regeneration with low driving force (Fig. 4), lower absorption in the visible range, the possibility to attain greater than 1 volt, and the possibility to exhibit high compatibility with alternative catalyst materials. These have achieved impressive photovoltaic performance under low light intensities, as indicated in numerous reports.^5,28,57–62^

**Fig. 5:**
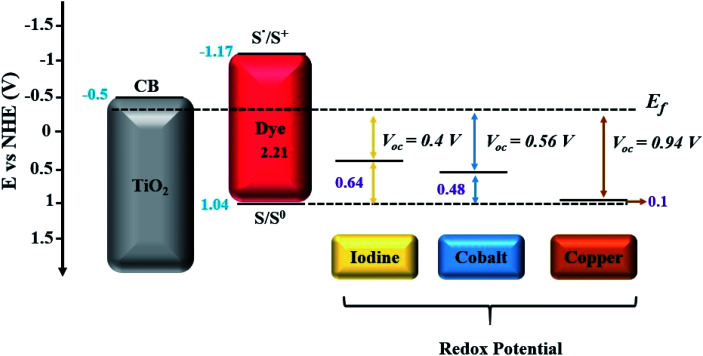
Energetics in DSSCs with respect to redox potentials of each redox couple (I^−^/I_3_^−^, [Co(bpy)_3_]^2+/3+^ and [Cu(dmp)_2_]^1+/2+^) utilized in DSSCs.^55^

Co complexes with tunable redox potential allow swift adjustments towards the HOMO level of the sensitizer, and ultimately offer the unique possibility for achieving higher open circuit voltages (Fig. 5).^55,57,63^ Among the few initial considerable demonstrations, Sandra *et al.* reported a 6.7% conversion efficiency of advanced DSSCs by selecting a suitable combination of a cobalt polypyridine complex and an organic sensitizer, which exhibited very high *V*_OC_ (>0.9 V) under full sun illumination.^57^ The same devices also exhibited striking photovoltaic performance (>7%) and outperformed iodine electrolyte containing DSSCs when measured under low light intensities. These promising results led to further testing of similar cobalt redox-based electrolyte systems with carbonaceous or polymer-based alternative catalyst materials, which showed promising compatibility and revealed very impressive 12–13% conversion efficiencies when measured under full sunlight illumination.^23,30^

Currently, lab-sized DSSCs employing [Co(phen)_3_]^2+/3+^ redox couple-based electrolyte formulations have surpassed >14% conversion efficiencies when tested with an alkoxysilyl-anchor dye (ADEKA-1) co-sensitized with a carboxy-anchor-based organic dye (LEG4) at AM 1.5 full sunlight irradiation. To date, this is the highest reported solar-to-electrical conversion efficiency with Co electrolytes during the development of next-generation DSSC devices.^24^

Nevertheless, despite the tremendous potential for achieving higher conversion efficiencies, the major limitations associated with the Co redox system include high recombination and mass transport limitations due to bulky ligands, which limits the short circuit current densities (*J*_SCs_) under full sunlight illumination.^64^

Similar to Co redox shuttles, the possibility of tuning the redox potential *via* ligand engineering of Cu redox complexes also makes it possible for improved redox potential matching with the HOMO level of the dye. Thus, higher *V*_OC_ (>1 V) can be achieved, while maintaining decent short current densities as indicated in many reports.^5,15,25,61^ As a result, impressive solar-to-electrical conversion efficiencies of DSSCs have recently been demonstrated with Cu electrolytes – not only under full sunlight illumination but also under low light intensity conditions – outperforming some of the other existing solar cell technologies.^2,5,15,62^

Moreover, the possibility of utilizing Cu redox shuttles as solid-state hole conductors also eliminates the leakage problem associated with liquid electrolytes and motivates the development of more robust DSSCs. Indeed, these may surpass the key stability tests for their reliable integration in both the BIPV and consumer electronic applications.^5,32,61^

Nevertheless, the current method of producing solid state DSSCs (ssDSSCs) – which involves introducing the Cu redox-based liquid electrolytes in the cell channel followed by evaporating the electrolyte solvent through drilled holes^5,32^ – seems impractical. This procedure must be done several times in order to create a solid mass of Cu redox complexes in between the PE and CE of the DSSC for working as a solid-state hole transporter.^5,32^ In terms of producing large area DSSC modules, this current scheme seems impractical not only from the economical point of view, but also because it can create additional cell resistance due to larger non-active areas in between the current collectors and active cell channel. Similarly, spatial variations can arise as a result of molecular filtering effects observed with iodide/triiodide redox-based electrolyte formulations in the first generation of DSSC devices.^39,65,66^

To address these challenges, inkjet printing appears to be a cost efficient and compatible materials deposition method (Fig. 2) for precise printing of these alternative solid-state Cu hole transporters over photoelectrodes, followed by mechanical pressing of PEDOT-coated CEs, as demonstrated by Cao *et al.*^25^ Even PEDOT catalyst layers could be inkjet-printed over these Cu redox-based advanced hole transporters to produce unique monolithic solid state DSSCs (discussed in Section 2). Moreover, the demonstration of inkjet printing of iodide/triiodide redox shuttle-based electrolytes has previously been reported for producing drilled hole-free DSSCs. In this case, enhancements in solar-to-electrical conversion efficiencies were observed as a result of eliminating the non-active area occupied by the drilled holes at the CE.^39^

Similar research on printable Cu redox-based solid-state hole conductors may be forecasted, which can result in producing not only fully printed lab-sized DSSCs, but may also lead to the development of large area based fully printed DSSC modules – one of the targeted goals of this promising photovoltaic technology. Table 1 summarizes some of the high efficiency DSSCs produced with Co- and Cu-based alternative electrolytes and solid-state hole conductors.

**Table 1:** Some of the high efficiency DSSCs produced with alternative Co and Cu redox based electrolytes and solid-state hole conductors

Electrolyte composition	Dye	PCE (%)	Stability	Year	Ref.
**Co electrolytes**					
0.20 M [Co^2+^(phen)_3_](PF_6_^−^)_2_, 0.05 M [Co^3+^(phen)_3_](PF_6_^−^)_3_, 0.07 M LiClO_4_, 0.02 M NaClO_4_, 0.03 M TBAPF, 0.01 M TBPPF, 0.01 M HMImPF, 0.30 M TBP, 0.10 M TMSP, 0.10 M MP, 0.05 M CPrBP, 0.10 M CPeBP, and 0.05 M COcBP in MeCN	ADEKA-1 + LEG4	14.3 @ 100 mW cm^−2^	Not reported	2015	24
		14.7 @ 50% mW cm^−2^			
(0.22 M [Co^II^(bpy)_3_](B(CN)_4_)_2_, 0.05 M [Co^III^(bpy)_3_](B(CN)_4_)_3_), 0.1 M LiClO_4_ and 0.85 M TBP in acetonitrile (ACN)	ZL003	13.6 @ 100 mW cm^−2^	50 days dark conditions with 25% RH 15% drop in efficiency was observed due to acetonitrile evaporation	2019	67
0.25 M Co(bpy)_3_(TFSI)_2_, 0.06 M Co(bpy)_3_(TFSI)_3_, 0.1 M LiTFSI, and 0.5 M 4-*tert*-butylpyridine in acetonitrile	SM342 + Y123	12.76 @ 100% sun intensity	Not reported	2017	30
		12.34 @ 10% sun intensity			
0.22 M Co(bpy)_3_(PF_6_)_2_, 0.05 M Co(bpy)_3_(PF_6_)_3_ (0.05 M), 0.1 M LiClO_4_, 0.2 M TBP, 0.1 M TPAA in acetonitrile	LEG4D35 + dyenamo blue	10.5 @ one sun illumination	Devices retained 89% of the initial efficiency when soaked in full sun light intensity up to 250 h and at 25 °C. Also, MPPT tracking was performed	2016	68
		10.2 @ 11.4 sun illumination			
		11.7 @ 0.46 sun illumination			
0.20 M [Co(bpy)_3_](TFSI)_2_, 0.06 M [Co(bpy)_3_](TFSI)_2_, 1.00 M *t*BP. 0.05 M LiTFSI in acetonitrile	MK2	9.42 @ 0.1 W cm^−2^	Not reported	2017	69

**Cu electrolytes**					
0.2 M Cu(tmby)_2_TFSI and 0.04 M Cu(tmby)_2_TFSI_2_, 0.1 M lithium bis(triuoromethanesulfonyl)imide and 0.6 M 4-*tert*-butylpyridine in acetonitrile or propionitrile	XY1:L1	11.5 @ full sun light intensity	16 h 1000 lux illumination at the daytime + 8 h of darkness for 12 days. Devices retained performance	2020	5
		34 @ 1000 lux intensity			
		32.7 @ 500 lux intensity			
		31.4 @ 200 lux intensity			
0.07 M Cu(i) and 0.05 M Cu(ii), 0.1 M LiTFSI, and 0.6 M TBP in acetonitrile	Y123	10.4 @ full sun intensity	Not reported	2020	61

**Cu(dmp)**_**2**_ **solid state HTM**					
0.06 M [Cu(tmby)_2_](TFSI)_2_, 0.2 M [Cu(tmby)_2_](TFSI), 0.1 M LiTFSI and 0.6 M TBP in acetonitrile	Y123	11.0 @ 1000 W m^−2^	Device stability of non-encapsulated cell was observed at ambient conditions which showed slight increase in the initial photovoltaic performance. Also, stability of one ssDSSCs operating at maximum output power was examined for 200 h under radiation at 500 W m^−2^, was examined *P*_max_ retains over 85% of its initial value	2017	70
		11.3 @ 500 W m^−2^			
		10.5 @ 100 W m^−2^			
0.2 M Cu(i) and 0.04 M Cu(ii) complexes and 0.1 M LiTFSI as well as 0.6 M TBP in acetonitrile or propionitrile	D35 + XY1	11.3 @ 100 mW cm^−2^	Not reported	2017	15
		25.5 @ 200 lux intensity			
		28.9 @ 1000 lux intensity			
0.10 M Cu(dmbp)_2_BF_4_, 0.05 M Cu(dmbp)_2_(BF_4_)_2_, 0.50 M TBP, and 0.10 M LiBF_4_ in acetonitrile	Y123	10.3 @ 100 mW cm^−2^	Stability test of one device was conducted for 15 days. 10% deviation in photovoltaic performance was observed due to acetonitrile evaporation in dark conditions	2017	71

## 4. Advanced TiO_2_ photoelectrodes

**Fig. 6:**
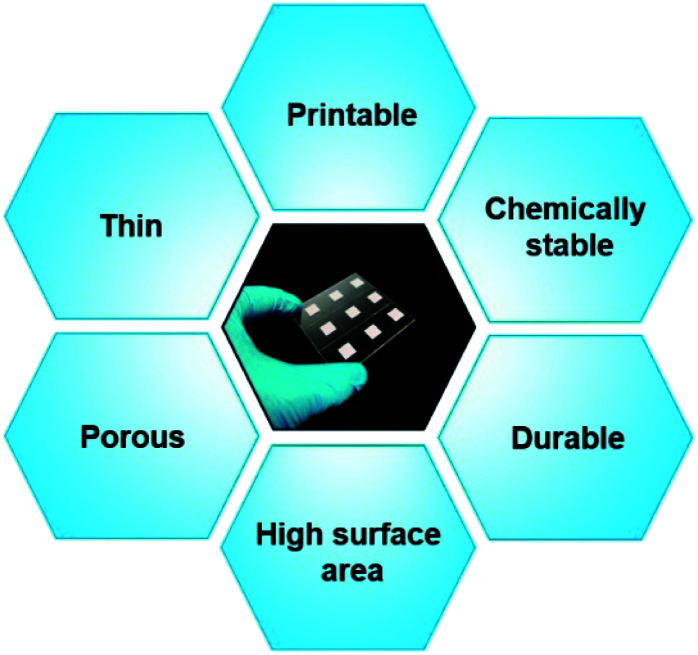
General characteristics of efficient semiconducting oxide layer in DSSCs.

Although many semiconducting oxides in DSSCs have been tested, the titanium dioxide (TiO_2_) nanoparticle-based electron transport layer proved to be the most efficient photoelectrode in the DSSC system, because of numerous characteristics (summarized in Fig. 6). In recent years, interesting trends and strategies have emerged where modified designs of traditional TiO_2_-based photoelectrodes^72^ have been proposed for achieving champion photovoltaic performances with advanced molecular light harvesters (dyes) and redox shuttles.^15,21,25,62^

For the first generation of DSSCs, typically 10–20 μm thick layers of TiO_2_ have been used with traditional ruthenium-based sensitizers to achieve high short circuit current densities (*J*_SC_).^72^ With TiO_2_ layers of such thickness, advanced redox couple-based electrolytes exhibit high diffusion resistance (*R*_D_)^28,30,73^ and limit the possibility of achieving higher *J*_SC_ than achieved with iodide/triiodide redox-based electrolytes.^72^

Nevertheless, the progress being made towards advanced light harvesters has resulted in the development of dyes with high absorption coefficients. These now make it possible to achieve similar *J*_SCs_ by sensitizing far thinner TiO_2_ photoelectrodes.^5,21,25,73^

Moreover, strategies such as porosity tuning of the porous TiO_2_ electrodes have also been reported with the Co redox-based liquid electrolytes. Titania films are produced with different sizes of TiO_2_ nanoparticles to avoid diffusion limitations of these bulky redox shuttles in the fabricated DSSC devices.^73^

More interestingly, the concentration adjustments of the widely adopted TiCl_4_ post-treatment method^74^ on these titania layers has also been proposed.^30^ This proved to have a systematic effect on *J*_SC_ values as a result of the influence on the TiO_2_ particle growth when tested with Co redox electrolytes.^30^ This seems logical in light of the improvements reported with iodide/triiodide redox based electrolytes that used standardized 40–50 mM concentration-based TiCl_4_ treatment methods,^74–76^ such as improved dye-loading and 10–30% increase in incident photon to collected electron efficiency (IPCE). Producing a gradient of TiCl_4_ solution concentration allowed the efficient tracking of the diffusion characteristics of bulky Co redox shuttles and was proven as an effective strategy to achieve higher *J*_SC_ values by suppressing diffusion resistances in the fabricated DSSCs.^73^ Such advanced and thinner TiO_2_ electrodes have contributed to impressive solar-to-electrical conversion efficiencies (∼12–13%) to date when tested with Co redox shuttle-based liquid electrolytes and porphyrin sensitizers.^21,23^

Such thinner and advanced TiO_2_ PEs have also been reported to produce DSSCs with alternative Cu redox shuttle-based electrolytes,^28,51^ where impressive solar-to-electrical conversion efficiencies under various light intensities are recently reported when tested with advanced light harvesters.^5,15,51,77^Table 2 highlights some of the top DSSCs produced with these advanced TiO_2_ PEs combined with various sensitizers, as well as Co and Cu redox shuttle-based alternative electrolytes.

**Table 2:** Some of the high efficiency DSSCs with TiO_2_ PEs combined with various sensitizers, as well as Co and Cu redox shuttle-based alternative electrolytes

Photelectrode (PE)	Thickness (μm), TiO_2_^a^ (nanocrystalline 20–40 nm), TiO_2_^a^ (scattering 200–400 nm)	TiCl_4_ conc. (mM), pre-treatment, post-treatment	Dye	Electrolyte composition	PCE^b^ (%)	Stability	Ref.
**With Co electrolytes**							
TiO_2_	3.5	Not reported	SM315	0.25 M Co(bpy)_3_(TFSI)_2_, 0.06 M Co(bpy)_3_(TFSI)_3_, 0.1 M LiTFSI, and 0.5 M 4-*tert*-butylpyridine in acetonitrile	13.0 @ 1000 W m^−2^	500 h @ full sun @ 298 K ∼25 °C no significant loss detected	23
	3.5	Not reported	SM371		12.0 @ 1000 W m^−2^		
TiO_2_	5	Not reported	YD2-o-C8/Y123	0.165 M Co^II^(bpy)_3_(B(CN)_4_)_2_, 0.045 M Co^III^(bpy)_3_(B(CN)_4_)_3_, 0.8 M *tert*-butyl pyridine (TBP), 0.1 M LiClO_4_ in acetonitrile	12.3 @ 99.5 mW cm^−2^	DSSC (unknown number of cells) were soaked in full sunlight at 30 °C for a period of 220 h, which led to 10–15% decrease in the overall efficiency	21
	5	Not reported			13.1 @ 50.8 mW cm^−2^		
					13 @ 9.4 mW cm^−2^		
TiO_2_	3.5	60 (twice)	SM342:Y123	0.25 M Co(bpy)_3_(TFSI)_2_, 0.06 M Co(bpy)_3_(TFSI)_3_, 0.1 M LiTFSI, and 0.25 M or 0.5 M 4-*tert*-butylpyridine in acetonitrile	12.76 @ 100% sun	Not reported	30
	3.5	20 mM			12.34 @ 10% sun		
TiO_2_	5	Not reported	Y123	0.2 M Co(bpy)_3_(B(CN_4_)_2_), 0.05 M Co(bpy)_3_(B(CN_4_)_3_), 0.1 M LiClO_4_, 0.2 M 4-*tert*-butylpyridine in acetonitrile	8.6 @ 100 mW cm^−2^	Not reported	73
	5	40			9.4 @ 50 mW cm^−2^		
					8.7 @ 10 mW cm^−2^		

**With Cu electrolytes**							
TiO_2_	4	Not reported	XY1:L1	0.2 M Cu(tmby)_2_TFSI, 0.04 M Cu(tmby)_2_TFSI_2_, 0.1 M lithium bis(trifluoromethanesulfonyl) imide, 0.6 M 4-*tert*-butylpyridine in acetonitrile	11.5 @ 100 mW cm^−2^	Not reported	5
	4	13			13.7 @ 10% sun		
					34 @ 1000 lux		
TiO_2_	4	40 (twice)	XY1 + 5T	0.2 M Cu^I^(tmby)_2_(TFSI), 0.06 M Cu^II^(tmby)(TFSI)_2_, 0.1 M lithium bis(trifluoromethanesulfonyl) imide (LiTFSI)_2_, 0.6 M *t*BP in anhyd acetonitrile	9.53 @ 100 mW cm^−2^	Not reported	78
	4	13			10.2 @ 10 mW cm^−2^		
					29.2 @ 1000 lux		
TiO_2_	3.5	40 (twice)	0.2 mM LEG4 in *tert*-butyl alcohol and acetonitrile	0.2 M Cu(dmp)_2_TFSI, 0.04 M Cu(dmp)_2_TFSI Cl, 0.1 M LiTFSI, 0.5 M 4-*tert*-butylpyridine (TBP) in acetonitrile	8.32 @ 96.515 mW cm^−2^	Not reported	51
	3.5	40			9.78 @ 50.791 mW cm^−2^		
					9.74 @ 12.279 mW cm^−2^		
TiO_2_	3	40	Y123	60.6 mg Cu(dmp)_2_TFSI, 13 mg Cu(dmp)_2_(TFSI)Cl, 12.6 mg LiTFSI 32 mg 4-tertbutylpyradine (4-TBP) in 0.4 ml of acetonitrile	7.0 @ full sun	Not reported	28
	Not reported	40			7.6 @ 50% sun		
					7.5 @ 10% sun		

a Particle size of TiO_2_ nano-particles.

b Champion device efficiencies.

## 5. New possibilities with inkjet-printed dyes

Development of novel dye designs for efficient light harvesting has always remained one of the major focus areas in DSSC research.^21,30,79^ However, less attention has been given to optimizing the traditional and time-consuming dye-sensitization process,^5,80^ which may limit the rapid production of large area DSSC modules.

**Fig. 7:**
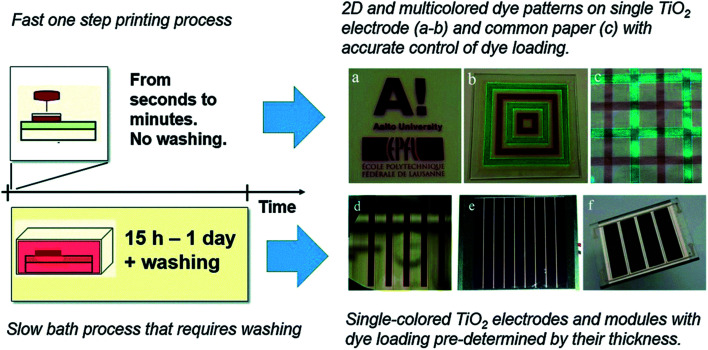
Rapidly sensitized photoelectrodes *via* inkjet printed dyes accelerates the staining process and can also be adopted to produce multicolour printed dyes based TiO_2_ electrodes and for precise co-sensitization of the PEs (reproduced from ref. 31 with permission).

In this regard, Hashmi and co-workers demonstrated rapid sensitization of TiO_2_ photoelectrodes *via* printing dye inks through a scalable and established inkjet printing method (Fig. 7).^38^ Considering the process where the dye molecules get adsorbed on the surface of the TiO_2_ particles, the inkjet printing is in principle a similar process as the conventional soaking process. The only difference is that in the inkjet printing, a much more concentrated dye solution is applied on the film, and the position where the solution soaks the film can be precisely controlled by the droplet deposition. It must be that the diffusion of the dye molecules inside the nanopores is much faster than the drying of the macroscopic droplet, and for this reason, the dye molecules have enough time to get absorbed on the walls of nanopores before the solvent evaporates.

This not only replaces the slow and dye bath-based conventional sensitization process of PEs, but also offers numerous opportunities. For example, multiple dyes can be printed with high precision over a solo TiO_2_ photoelectrode, which enables for the first time the possibility to create a variety of colourful patterns, and greatly motivates the development of colourful photographs similar to digital pictures as functional solar cells.

In addition, this method allows control over the transparency of a single TiO_2_ electrode through depositing different amounts of dye in different parts of the electrode Previously, tuning the transparency of the DSSC has been possible only in a spatially uniform manner, by either adjusting the thickness of the TiO_2_ layers or the conditions in the dye bath process (concentration, duration, temperature and pressure).^81–83^ The spatial control of the dye loading of one or more dyes provides an interesting opportunity to design digitally printed colour patterned DSSCs for use in design and architecture.

Furthermore, inert environmental conditions for sensitizing TiO_2_ electrodes may also be avoided if executed through the inkjet sensitization scheme. During the inkjet printing step, the dye ink remains preserved in the sealed cartridge and gets deposited from nozzles with a very small drop volume (1 or 10 picolitre), which minimizes the risk of direct exposure to air and humidity. As a result, this contaminant-free dye solution can surely facilitate in achieving long-term photovoltaic performance stability of fabricated DSSCs in various stressful environmental conditions.^38^

In general, executing the sensitization step with inkjet printing technology brings new possibilities, which may also influence the overall manufacturing cost and the photovoltaic performance reproducibility in understanding the production of fully printable next-generation DSSC technology (Fig. 4). Nevertheless, one possible drawback associated with inkjet printing of dyes is the limited chemical compatibility of the dye solvents with the nozzles of the cartridge print head. The use of harsh solvent-based dyes could possibly trigger unwanted chemical reactions, which may block the nozzles of the cartridge print head and affect the precision of dispensing the targeted amount to sensitize the TiO_2_ layers. Although there are several successful demonstrations of inkjet printing of concentrated dye solutions with dimethylformamide (DMF) and dimethyl sulfoxide (DMSO) solvents reported,^38^ more studies are needed to investigate other compatible solvents that could also facilitate the inkjet printing of the dye step during the production of next generation-based printable DSSCs.

## 6. Progress in catalysts research

In addition to the advancements being made in electrolytes and photoelectrodes research, an interesting shift in determining standard catalyst materials to be used with novel redox shuttles has also emerged.^5,28,35^

For the first generation of DSSCs, a platinum (Pt) catalyst has remained the first choice in producing high efficiency DSSCs.^20,21,74^ Being highly catalytic and exhibiting robust mechanical durability and chemical stability, Pt CE-based DSSCs have thus far demonstrated >11.0% solar-to-electrical conversion efficiency when tested with iodide/triiodide redox shuttle-based electrolytes.^20^ Moreover, Pt CE-based DSSCs have also exhibited robust stability by showing no performance loss when subjected to long-term stability tests under various simulated stability conditions.^54,84^

Unlike Pt, which does not have sufficient catalytic activity for use with the Co- and Cu-based redox couples,^28,59,85,86^ electro-deposited PEDOT has shown good catalytic performance. Indeed, high efficiencies have been reached both at high and low light intensities with these new redox couples.^5,15,25^ Nevertheless, the chemical stability of the PEDOT catalyst layer has yet to be determined since the long-term stability of the DSSC-employed PEDOT catalyst and Cu redox shuttles is rarely reported.^5,70^ This raises the concerns of long-term and stable photovoltaic operation of these next-generation DSSCs when targeted to either portable electronics or IoTs as indoor applications, or even when exposed to natural climatic conditions for bulk electricity generation outdoors.

In a recent study, stable charge transfer resistance (*R*_CT_) over a longer period (2000 h) of PEDOT catalyst-based CEs was observed in DSSCs when fabricated with an organic dye (Y-123) and cobalt (Co) electrolytes.^35^ Nevertheless, electrolyte degradation was suspected as a result of a gradual increase in diffusion resistance (*R*_D_), which consequently affected the short (*J*_SC_).^35^ Hence, further advancement towards understanding the electrochemical behaviour in order to improve chemical stability of the PEDOT catalyst is highly expected in the near future.

**Fig. 8:**
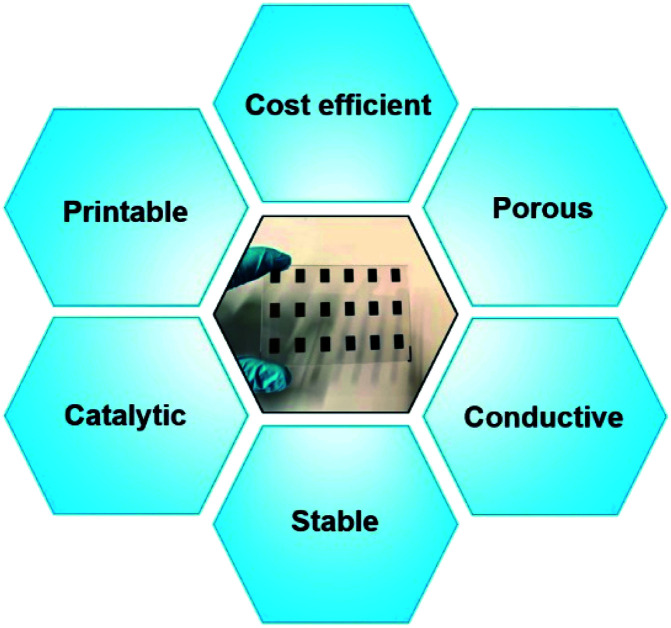
General characteristics of an efficient carbonaceous counter electrode in DSSCs.

On the other hand, low cost carbonaceous catalyst materials have also been proven as another suitable alternative catalyst material for both Co and Cu redox shuttle-based electrolytes,^21,28,59,85–88^ owing to their numerous characteristics such as high surface area, high porosity and high catalytic activity along with possibility of their precise printing (Fig. 8). Impressive solar-to-electrical conversion efficiencies have been frequently demonstrated^21,87^ and have exceeded over 14% when employing carbonaceous counter electrodes, thus surpassing the previous efficiency records achieved with traditional Pt catalyst layer-based CEs.

Among such carbonaceous materials, graphene nanoplatelets have mostly been tested with Co and Cu redox shuttle-based electrolytes. These outperformed traditional Pt catalysts by showing high catalytic activity, and by exhibiting lower *R*_CT_ during electrochemical characterizations of reported DSSCs.^59,87,88^

Interestingly, one of the current limitations associated with both the tested electro-deposit PEDOT and graphene nanoplatelets is their materials deposition method (as demonstrated for lab-sized DSSCs),^5,59^ which cannot be used to realize grid-type large area modules production since they cannot be precisely patterned. This raises concerns for both the scaling and the photovoltaic performance reproducibility within their production batches.

Keeping such limitations in mind, Hashmi *et al.* recently demonstrated printable single-walled carbon nanotube (SWCNT)-based CEs as a more practical approach^28^ to addressing the challenge regarding scalable production of DSSC with advanced sets of materials. Similar to graphene nanoplatelets, these printed SWCNT-based CEs also outperformed the traditional Pt nano catalyst by revealing significantly lower *R*_CT_ (2–2.9 Ω cm^2^) when loaded with Cu redox shuttle-based electrolytes. They also contributed to achieving very impressive solar-to-electrical conversion efficiencies, for example 7.5% and 8.3%, when measured under full and half sun illumination, respectively.^28^

Such promising results also motivate more economical approaches to be adopted in the future. For example, such SWCNTs CEs could be replaced with further low-cost composites of carbon black and graphite nanoparticles, which have frequently been reported to produce both traditional and monolithic DSSCs with iodide/triiodide redox-based electrolytes.^19,89,90^ Although no such studies are published thus far, these low-cost and printable carbon composites are expected to be tested with Cu redox shuttle-based electrolytes to produce scalable DSSC modules as futuristic advancements in DSSC research. Table 3 summarizes the best and most recent DSSC efficiencies achieved by employing alternative catalyst materials along with novel Co and Cu redox shuttle-based electrolytes.

**Table 3:** Champion DSSCs reported in recent years employing alternative catalyst materials

Catalyst (CE)	Electrolyte composition	*R*_CT_ a (Ω cm^2^)	PCE (%)	Stability	Ref.
**With Co electrolytes**					
Graphene	0.22 M Co(bpy)_3_(PF_6_)_2_, 0.05 M Co(bpy)_3_(PF_6_)_3_, 0.1 M LiClO_4_, and 0.2 M 4-*tert*-butylpyridine in acetonitrile	0.2	9.4 @ 1 sun	Not reported	59
			9.6 @ 0.51 sun		
			9.3 @ 0.095 sun		
Carbon	0.6 M [Co(phen)_3_]^2+/3+^ (Co(ii)/Co(iii) ratio of 4 : 1), 0.15 M LiTFSI, and 0.8 M TBP in acetonitrile	2.92	9.53 @ 100 mW cm^−2^	Not reported	91
			10.03 @ 50 mW cm^−2^		
			9.21 @ 10 mW cm^−2^		
Selenide/graphene composite	0.21 M [Co(bpy)_3_](TFSI)_2_, 0.068 M [Co(bpy)_3_](TFSI)_3_, 0.95 M tBP, and 0.055 M LiTFSI in ACN	4.13	11.26 @ 100 mW cm^−2^	Stability of one DSSC reported for >336 h. The device was stored in dark at 25 °C	19
Graphene nanoplatelets	0.25 M [Co(bpy)_3_][B(CN)_4_]_2_ and 0.06 M [Co(bpy)_3_][B(CN)_4_]_3_ complexes with 0.1 M LiTFSI and 0.5 M 4-*tert*-butylpyridine in acetonitrile	∼30^a^	10.3 @ 1 sun	Not reported	92

**With Cu electrolytes**					
SWCNT	60.6 mg Cu(dmp)_2_TFSI, 13 mg Cu(dmp)_2_(TFSI)Cl, 12.6 mg LiTFSI, and 32 mg 4-*tert*-butylpyradine (4-TBP) in acetonitrile	∼2.1–2.9	7.5 @ full sun illumination	Not reported	28
			8.3 @ half sun illumination		
PEDOT	0.2 M Cu(i) and 0.04 M Cu(ii) complexes and 0.1 M LiTFSI as well as 0.6 M TBP in acetonitrile or propionitrile	Not reported	11.3 @ 100 mW cm^−2^	Not reported	15
			25.5 @ 200 lux intensity		
			28.9 @1000 lux intensity		
PEDOT	0.2 M Cu(tmby)_2_TFSI, 0.04 M	Not reported	11.5 @ full sun intensity	16 h 1000 lux illumination at the daytime + 8 h of darkness for 12 days. Devices retained photovoltaic performance	5
	10 Cu(tmby)_2_TFSI_2_, 0.1 M lithium bis(trifluoromethanesulfonyl)imide, and 0.6 M 4-tertbutylpyridinein acetonitrile		34 @ 1000 lux intensity		
			32.7 @ 500 lux intensity		
			31.4 @ 200 lux intensity		
PEDOT	0.04 M [Cu(tmby)_2_](TFSI)_2_, 0.20 M [Cu(tmby)_2_]TFSI, 0.1 M LiTFSI, and 0.6 M 1 methylbenzimidazole in acetonitrile	Not reported	13.1 @ 100 mW cm^−2^	DSSC remained stable (4 days) when stored in ambient dark conditions. DSSC retained 90% of its initial value during maximum power (*P*_max_) point tracking for 10 h under 100 mW cm^−2^ continuous light soaking at 45 °C	25
			13.1 @ 50 mW cm^−2^		
			12% @ 10 mW cm^−2^		
			31.8 @ 1000 lux		
			30.8 @ 500 lux		
			27.5 @ 200 lux		

**Cu(dmp)**_**2**_ **solid state HTM**					
PEDOT	0.06 M [Cu(tmby)_2_](TFSI)_2_, 0.2 M [Cu(tmby)_2_](TFSI), 0.1 M LiTFSI and 0.6 M TBP in acetonitrile	Not reported	11.0 @ 1000 W m^−2^	Device stability of non encapsulated cell were observed at ambient conditions which showed slight increase in the initial photovoltaic performance. Also, stability of one ssDSSCs operating at maximum output power was examined for 200 h under radiation at 500 W m^−2^, was examined *P*_max_ retains over 85% of its initial value	70
			11.3 @ 500 W m^−2^		
			10.5 @ 100 W m^−2^		

a The values were evaluated from the curves in and the unit is Ω.^92^

## 7. Advanced sealing techniques

In addition to the continuing efforts being made towards improving solar-to-electrical conversion efficiencies of DSSCs, several conventional approaches, or those that use innovative encapsulation materials, were employed to produce stable and robust PV devices.^39,53,54,84^

The encapsulation of DSSCs involves appropriate design considerations to maintain high performance and economy of scale. To ensure long-term stability and reliability, durable sealing materials and procedures are mandatory for protecting the active geometry of the DSSCs from external factors.^11,29,93^ To address such challenges, the sealant must withstand the changing environmental conditions during the DSSC's lifetime. It should provide strong mechanical support to resist external and internal strains that could damage the active components of the DSSCs.

**Fig. 9:**
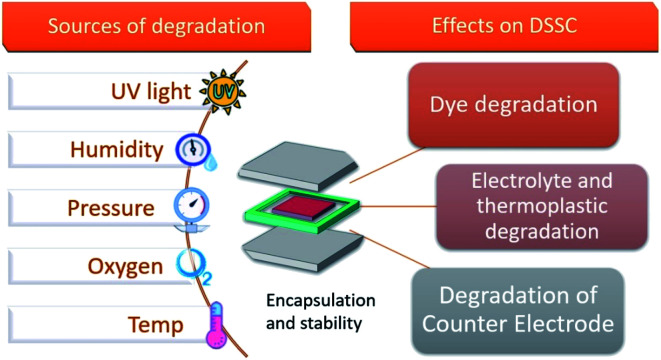
Illustration of the factors that affect DSSC devices and their possible consequences which hinder the photovoltaic performance.

The typical degradation factors (Fig. 9) reported for DSSCs include intrusion of moisture and oxygen in the cell active area, electrolyte sensitivity towards UV light, electrolyte leakage and electrolyte solvent evaporation when subjected to stressful climatic and simulated environmental conditions.^11,93–95^

Among these issues, the sealing procedures for isolating liquid electrolytes in cell channels is challenging.^11,52^ Regardless of the recent literature and evidences for stable PV performance,^38,39,53,54,84^ the long-term operational stability of DSSCs has been subjected to reservation mainly due to electrolyte leakage from the cell channels. This has been considered as one of the bottlenecks for the successful commercialization and widespread use of this promising PV technology.^52,95^

Nevertheless, by taking advantage of glass as a robust substrate, glass–glass encapsulations (by utilizing either thermoplastic or glass frit-based sealings^38,39,81^) or glass thermoplastic sealing procedures were adopted for various DSSC device designs to achieve notable stability under natural and simulated environmental conditions.^27,38,39,53,54,80,83,89,96^

Moreover, contrary to conventional thermoplastics (*i.e.* Surlyn or Bynel), solutions such as impermeable sealing to eliminate oxygen and water intrusion, and to avoid metal finger corrosion, seem inevitable for allowing DSSCs commercialization for large modules in the coming years.^97^

In this direction, new protocols for isolating liquid electrolytes in the cell channel are being exploited,^5,25^ in which both the directly contacted PE and CE are glued together, illustrated in the pioneering work by Cao *et al.* (discussed in Section 2) using UV-curable glue to isolate the liquid electrolyte.^25^ Following the electrolyte injection *via* drilled holes, it is then sealed with the same UV-curable glue. The sealed device exhibited preliminary short-term (10 hours) stability when exposed to full sunlight intensity under thermal stress of 45 °C.^25^

Further advancement was recently shown by Hannes *et al.*; a device design similar to that reported by Cao *et al.* was demonstrated, but with improved PV performance. Here the electrodes were sealed under similar UV-curable glue, followed by a slight modification at the end of the encapsulation scheme, *i.e.* the drilled hole was sealed with traditional thermoplastic and a glass cover slip (as in traditional DSSCs sealing) after electrolyte injection.^5^ The devices exhibited stable and long-term PV performance when tested for powering up an IoT under low light (1000 lux) intensity indoors at 16 hours day for a period of 12 days under room temperature conditions.^5^

Though these approaches and preliminary reports look promising, the aforementioned novel sealing method may not be opted for yet as the standard protocol for DSSCs sealing, since further testing under more stressful conditions (*i.e.* higher temperature ranges: 60–80 °C combined with full sunlight soaking) is still needed to confirm its extreme limits of robustness. Additionally, this manual sealing method also seems impractical for producing such mechanically pressed PE- and CE-based series or parallel connected DSSC modules. The conventional thermoplastics (such as Surlyn or Bynel) are typically used for defining series of parallel connected individual cell fingers in two substrate-based grid type DSSC modules.^98,99^

Nevertheless, the UV-curable glue may be directly screen-printed over serially connected active layers for producing robust and stable solid-state novel monolithic device design with a printable solid-state hole conductor of Cu redox shuttles (discussed in Section 2). Table 4 briefly highlights some of the sealing materials used to produce DSSCs with promising stability in recent years.

**Table 4:** DSSCs reports produced with several sealing materials

Device design	Sealant	Stability	Ref.
FTO glass PE – FTO glass CE	Surlyn	Stability of one DSSC reported, which retained 90% of initial photovoltaic performance for 500 hours under continuous full sun irradiation with a UV cut-off filter	100
FTO glass PE – FTO glass CE	Surlyn, bynel and epoxy	Champion DSSCs with ACN maintained 66% of the initial efficiency after 2000 hours at 20 °C and 1 sun light intensity. DSSCs with 3-MPN solvent based electrolyte maintain 91% of the initial efficiency	101
		DSSCs with ACN solvent based electrolyte maintain 100% efficiency at maximum power point at 30 °C for a period of 1000 h	
FTO glass PE – FTO glass CE	“Surlyn and hermetic sealing with epoxy adhesive (3 M)”	DSSC maintained 90% of initial PCE with full sunlight soaking at 60 °C for 1000 h	102
FTO glass PE – FTO glass CE	Surlyn	The QS-DSSCs with dyes N-719 and Z-907 retained 95% and 97% of their initial value under continuous light illumination of 200 lux at 35 °C after 1000 h	80
FTO glass PE – FTO glass CE	UV curing glue	Stability of one DSSC reported, which retained 92% of its peak value during light soaking test at 60 °C for 500 h	103
Thin film (AlO_*x*_N_*y*_) coated polymer (PEN) polymers were used as substrates	The PE and CE were attached with epoxy resin	DSSC retained 50% of the initial value after 300 h	104
FTO glass PE – FTO glass CE	UV curing glue	DSSC remained stable (4 days) when stored in ambient dark conditions. DSSC retained 90% of its initial value during maximum power (*P*_max_) point tracking for 12 h under 100 mW cm^−2^ continuous light soaking at 45 °C	25

## 8. New opportunities for standardizing stability testing protocols of novel DSSCs for indoors applications and IoT devices

Currently, the deployment of DSSCs outdoors or their integration in building integrated photovoltaics (BIPV) applications requires reliable certifications for their long-term photovoltaic performance stability under severely stressful conditions.^105,106^ On the other hand, the forecasted deployment of next-generation DSSCs in IoT devices as efficient energy harvesting units indoors^5^ may relax the certification conditions, which could consequently lead to a commercial breakthrough. This is mainly due to different ecological conditions inside modern buildings, which not only maintain controlled environments^107^ but also remain far less stressful than simulated^39,54,108^ or natural climatic conditions outdoors.^109^ Mindful of the need for standardizing the set of testing protocols for the next generation of photovoltaic technologies based on emerging organic solar cells,^110^ DSSCs^29^ or perovskite solar cells,^8,9,111^ consensus statements^105,106^ have been recently reported to provide guidance for their reliable testing procedures and conditions for converting lab-sized solar cells into reliably integrable commercial products.^14,112,113^ With such previous practices, new consensus statements for standardizing new testing protocols seems logical, and can be predicted for various reasons, such as:

(1) The abundance of numerous light sources (including fluorescent lights, LEDs, sodium and halide lamps available with a wide variety of spectrums) that have been installed in modern buildings. This makes an interesting situation for the prime selection of standard light sources for determining the reliable conversion efficiencies.

(2) Determining the standard temperature ranges for thermal stress testing, since the room temperature conditions remain far lower compared to the most demanding test (*i.e.* 85 °C combined with 85% RH)^105,106^ needed to surpass in order to install the devices under natural climatic conditions outdoors. Nevertheless, the selection of temperature ranges for obtaining indoor installation certificates could remain influential from the perspective of transportation and storage of the fabricated solar cells or modules, where they could experience a wide range of temperatures before their final installations at the selected sites.

(3) An updated UV stress test for these advanced DSSCs could be adopted with a slight relaxation compared to the stressful UV stability tests,^11,114,115^ aimed for their indoor deployment under modern LED light sources. These LED light sources have been widely deployed in current buildings as a low-cost, stable and energy efficient alternative to traditional filament-based light sources, and do not contain UV in their light spectrum. Therefore, such LED light sources gives a great possibility to next-generation DSSCs for long-lasting and efficient power generation under their irradiation for longer periods if integrated in futuristic IoT devices.

(4) A possible reform in the traditional 1000 hours (6 weeks) of continuous stability tests^38,39,54,84^ may also be realized by further extending the exposure time to 2000 hours, since the rate of chemical reactions within the DSSCs could be far slower due to the less stressful conditions indoors.

Hence, all these interesting possibilities motivate the development of a special set of stability tests to assess the reliable potential of next-generation DSSC devices to be operated under far more relaxed conditions than those used outdoors. Table 5 suggests several potential stability tests that may be adopted from previously established ISOS testing protocols^105^ to assess the photovoltaic performance stability of these next-generation DSSC devices, for their deployment as energy harvesting units in the futuristic IoT devices and portable electronics.

**Table 5:** Few proposed stability test protocols for indoor testing^a^

Test id	Light source	Temperature	Rel. humidity	Environment/set-up	Characterization light source	Load
**Dark storage (ISOS-D)**						
ISOS-D-1^b^	None	RT	Ambient	Ambient air	Indoor light source	OC
ISOS-D-2^c^	None	60 °C	Ambient	Oven, ambient air	Indoor light source	OC

**Bias stability (ISOS-V)**						
ISOS-V-1^b^	None	RT	Ambient	Ambient air	Indoor light source	Positive:VMPP; *V*_oc_

**Light-soaking (ISOS-L)**						
ISOS-L-1^b^	Indoor light source	RT	Ambient	Light only	Indoor light source	MPP or OC
ISOS-L-2^c^	Indoor light source	60 °C	Ambient	Light and temperature	Indoor light source	MPP or OC

**Thermal cycling (ISOS-T)**						
ISOS-T-1^c^	None	RT to 60 °C	Ambient	Hot plate/oven	Indoor light source	OC
ISOS-T-2^b^	None	RT to 65 °C	Ambient	Oven/env. chamber	Indoor light source	OC
ISOS-T-3^c^	None	−40 to +65 °C	<55%	Env. chamber	Indoor light source	OC

**Light cycling (ISOS-LC)**						
ISOS-LC-1^b^	Indoor light source/dark cycle	RT	Ambient	Light only	Indoor light source	MPP or OC

a Reported table is an alternation from a Table 1 presented in ref. 105.

b Original test.

c Modified/recommended test ISOS standards have solar simulator or sunlight for light source, which are not suitable for indoors stability testing and have been changed to indoor light source *V*_oc_, *V*_MPP_ are determined from light *J*–*V* curves^105^ RT = room temperature 23 ± 4 °C, RH = relative humidity, OC = open-circuit condition, MPP = maximum power point.

## 9. Summary and conclusions

Dye-sensitized solar cells (DSSCs) are efficient in generating the energy required for electronic applications such as wireless sensors, though harvesting indoor lighting. Their inexpensive and abundant materials, along with our ability to fabricate them as thin and light-weight flexible solar panels makes them well-suited for producing low-cost indoor solar panels, provided that the cell manufacturing methods can be scaled to industrial production with high cell efficiency and long-term indoor durability. The research trends discussed in this work related to the production of next-generation DSSCs show that important progress has been made with new and optimized materials, ultimately increasing the photovoltaic performance of these photovoltaic devices. These alternative materials offer new possibilities for fabricating advanced DSSC designs such as mechanically contacted liquid junction or solvent free solid-state zombie DSSCs. Moreover, the process flow suggested in this work offers a more economical approach to producing an advanced DSSC device structure on a single glass substrate, which may significantly influence the overall production costs. Producing solid-state DSSC architecture with the suggested process flow on single substrates with printed dyes and a Cu redox based solid-state hole conductor will further increase the robustness of DSSCs under natural and simulated environmental conditions, and will provide new opportunities for portable electronics and internet-of-things devices. Therefore, rapid research and development activities from many research labs and commercial players can be forecasted, which may accelerate the vast spread of DSSC technology at an affordable cost and with influential socio-economic impact.

## Author contributions

J. Z. and S. A. contributed to Sections 3 and 6, P. T., M. K. contributed to Sections 2, 3 and 8, S. S. and F. E. contributed in Section 7 with scientific writing, illustrations and tables drafting. J. H., S. A. and A. H. contributed with overview and comments on the manuscript. The authors thank Jacquelin De Faveri for proof-reading the manuscript. G. H. supervised the research work and contributed with funding acquisition, outline drafting, reviewing, and editing the overall text, tables and illustrations of the manuscript.

## Conflicts of interest

The authors express no conflict of interest in between them.
